# An analysis of published cases of cutting balloon use in spontaneous coronary artery dissection

**DOI:** 10.3389/fcvm.2023.1270530

**Published:** 2023-11-09

**Authors:** Bojan Maričić, Zoran Perišić, Tomislav Kostić, Nenad Božinović, Milovan Petrović, Milenko Čanković, Zlatko Mehmedbegović, Stefan Juričić, Vladimir Vasilev, Sonja Dakić, Jelena Perišić, Jelena Milošević, Mihajlo Bojanović, Miroslav Nikolić, Tijana Maričić, Svetlana Apostolović

**Affiliations:** ^1^ Department of Cardiology, University Clinical Center Nis, Niš, Serbia; ^2^ Faculty of Medicine, Department of Internal Medicine, University of Nis, Niš, Serbia; ^3^ Department of Cardiology, Institute for Cardiovascular Diseases of Vojvodina, Sremska Kamenica, Serbia; ^4^ Faculty of Medicine, Department of Internal Medicine, University of Novi Sad, Novi Sad, Serbia; ^5^ Department of Cardiology, University Clinical Center Belgrade, Belgrade, Serbia; ^6^ Faculty of Medicine, Department of Internal Medicine, University of Belgrade, Belgrade, Serbia; ^7^ Department of Cardiology, VFV Clinic Skopje, Skopje, North Macedonia; ^8^ Department of Anesthesiology, Resuscitation and Intensive Therapy, University Clinical Center Nis, Niš, Serbia

**Keywords:** cutting balloon, spontaneous coronary artery dissection, acute coronary syndrome, angioplasty, intramural hematoma

## Abstract

**Introduction:**

SCAD involves a sudden tear or separation within the layers of the coronary artery wall, resulting in blood flow obstruction and subsequent myocardial ischemia.

**Materials and methods:**

A comprehensive literature search was conducted to identify relevant published cases of cutting balloon use in patients diagnosed with spontaneous coronary artery dissection. Electronic databases including PubMed, MEDLINE, Embase, Cochrane Library and Google Scholar were systematically searched from inception until the present using terms “cutting balloon,” “SCAD,” “acute coronary syndrome,” “intramural hematoma,” and “angioplasty.”

**Results:**

A total of 32 published cases of cutting balloon use in spontaneous coronary artery dissection were analyzed in this study. The majority of the patients included in the analysis were female without prior history of cardiovascular disease. The median age of the SCAD population was approximately 46 years. The most frequently affected artery in SCAD cases was the Left Anterior Descending artery. Intravascular ultrasound was utilized more frequently than other modalities of adjunctive imaging techniques. The most frequent complication was the distal propagation of hematoma. Despite the successful dilation achieved with the cutting balloon, in some cases stenting was required to provide additional support.

**Conclusion:**

The results of this analysis demonstrate that cutting balloon use in SCAD cases yields favorable outcomes.

## 1. Introduction

Spontaneous coronary artery dissection (SCAD) is a rare but increasingly recognized cause of acute coronary syndrome, particularly in young women without traditional cardiovascular risk factors. SCAD involves a sudden tear or separation within the layers of the coronary artery wall, resulting in intramural hematoma formation, blood flow obstruction and subsequent myocardial ischemia (Supplementary Figure 1) (1).

SCAD can be classified based on angiographic findings as (2):

• Type 1 (an obvious stain on the wall of the artery with the presence of a double lumen)
• Type 2 (diffuse smooth stenosis of varying degrees, usually >20–30 mm)
• Type 3 (focal or tubular stenosis mimicking atherosclerosis usually 11–20 mm)
• Type 4 (dissection leading to a sudden total occlusion, usually of the distal coronary segment)

Among the evolving treatment modalities, the utilization of cutting balloons has garnered significant attention as a potential intervention in SCAD cases. Cutting balloons, initially designed for angioplasty procedures, employ microsurgical blades mounted on the surface of a balloon to incise and dilate the affected arterial segment. This unique mechanism offers potential benefits in dissection management by creating controlled micro-incisions within the affected vessel, causing intimal fenestration and hematoma draining (Supplementary Figure 2) (3, 4).

While the use of cutting balloons in SCAD treatment has gained some clinical traction, the body of evidence supporting its efficacy and safety remains limited. Published cases reporting the application of cutting balloons in SCAD cases offer valuable insights into the procedural aspects, outcomes, and potential benefits or drawbacks associated with this approach (5, 6).

Therefore, this paper aims to perform a comprehensive analysis of published cases of cutting balloon use in SCAD. By synthesizing existing data, we intend to evaluate the clinical outcomes, technical considerations, and potential complications associated with the utilization of cutting balloons in this unique patient population.

The findings from this analysis have the potential to enhance our understanding of the role of cutting balloons in SCAD management and guide clinical decision-making in treating this challenging condition. As SCAD remains an underdiagnosed and understudied entity, this paper aims to contribute to the growing body of literature on novel therapeutic strategies, further advancing the field and ultimately improving patient outcomes.

## 2. Materials and methods

A comprehensive literature search was conducted to identify relevant published cases of cutting balloon use in patients diagnosed with SCAD. Electronic databases including PubMed, Embase, and Cochrane Library were systematically searched from inception until the present, with no language restrictions. The search strategy involved a combination of controlled vocabulary terms (MeSH terms) and keywords related to “cutting balloon,” “SCAD,” “acute coronary syndrome,” “intramural hematoma,” and “angioplasty.” Additionally, reference lists of included studies and relevant review articles were manually screened for additional eligible cases.

The extracted data were tabulated and qualitatively analyzed to identify patterns, trends, and potential associations. Descriptive statistics, including frequencies and percentages, were used to summarize categorical variables, while continuous variables were reported as means or medians with corresponding measures of variability.

## 3. Results

A total of 32 published cases of cutting balloon use in SCAD were analyzed in this study (7–32). The majority of the patients included in the analysis were female (*n* = 30, 93.75% female, *n* = 2, 6.25% male) and had no prior history of cardiovascular disease (CVD). The median age of the SCAD population was approximately 46 years (28–73 years). The most frequently affected artery in SCAD cases was the Left Anterior Descending (LAD) artery, observed in *n* = 24, 75% of the cases.

Overall, the procedural outcomes of cutting balloon use in SCAD were encouraging. The majority of cases resulted in a Thrombolysis In Myocardial Infarction (TIMI) 3 flow restoration (*n* = 30, 93.75% of cases) (Supplementary Table 1). This indicates successful reperfusion and optimal blood flow through the affected coronary artery.

In terms of adjunctive imaging techniques, intravascular ultrasound (IVUS) was utilized more frequently than other modalities. IVUS was employed in *n* = 53.12% of the cases, providing detailed information about the extent and characteristics of the dissection and helping guide the cutting balloon intervention.

In some cases, additional treatment was necessary after cutting balloon angioplasty. Stenting was performed as a follow-up intervention in *n* = 12, 37.5% of the cases (Supplementary Table 1). This suggests that despite the successful dilation achieved with the cutting balloon, stenting may be the final option to provide additional support and stabilize the dissected coronary artery.

It is worth noting that the specific outcomes related to procedural success, TIMI flow, and the need for additional interventions may vary depending on individual patient characteristics, severity of SCAD, and the expertise of the operators.

The diameter of the cutting balloons used in the analyzed cases was consistently smaller than the vessel diameter, with the most common size being 2.5 mm (*n* = 11, 35.49%) (Supplementary Table 1). This approach of using a smaller cutting balloon size compared to the vessel diameter aimed to minimize the risk of vessel injury and optimize the efficacy of the procedure (Supplementary Figure 2).

Among the reported complications, the most frequently encountered was the distal propagation of the subintimal hematoma. This complication occurred in *n* = 6, 18.75% of the cases and highlights the importance of careful monitoring and management during and after CB angioplasty and stenting.

The deployment of the cutting balloon was primarily carried out at the level of the maximal lumen compression (Supplementary Figure 2). If there was no visual improvement, distal inflations were done. This approach allowed for precise positioning of the cutting balloon and focused dilatation within the affected segment. By targeting the lesion directly, the cutting balloon intervention aimed to effectively modify the dissected arterial segment while minimizing unnecessary trauma to the surrounding healthy tissue.

Furthermore, the majority of patients included in the analysis presented with ST-elevation myocardial infarction (STEMI) *n* = 10, 31.25%. This suggests that SCAD, particularly when involving the LAD artery, can lead to severe ischemic events requiring urgent intervention. The utilization of cutting balloons in these STEMI patients aimed to promptly restore blood flow and salvage viable myocardium.

Overall, the results of this analysis demonstrate that cutting balloon use in SCAD cases, particularly among female patients with no prior history of CVD, yields favorable outcomes. However, further research is warranted to explore the long-term clinical implications, patient prognosis, and compare the effectiveness of cutting balloon angioplasty to other treatment approaches in SCAD management.

## 4. Discussion

The management of SCAD remains a challenging clinical scenario due to its unpredictable presentation and potential for catastrophic outcomes. In recent years, cutting balloons have emerged as a potential therapeutic option for SCAD, offering a unique approach to dissection management.

Our analysis revealed several key findings that contribute to the existing knowledge base on the role of cutting balloons in SCAD management. Firstly, we observed that stenting was employed as an adjunctive therapy rather than a standalone treatment modality (Supplementary Table 1) (8, 13, 15, 16, 18, 20, 27, 28, 29, 31, 32). In all of the cases where stenting was performed, it was utilized in combination with cutting balloon angioplasty. This suggests that cutting balloons may serve as a useful tool in the armamentarium of SCAD treatment, augmenting the effects of conventional therapies.

Although majority of cases did not have a previous history of cardiovascular diseases *n* = 7, 21,87%, some cases described SCAD during pregnancy or early postpartum, which can be consider as provoking state. [Macaya, Matsuura, Mailey, Somerville, Ejima, Low]. Hormonal and hemodynamic changes during pregnancy can provoke SCAD. Increase in sympathetic activity and activation of the renin-angiotensin-aldosterone system with increased cardiac output, blood volume and red cell mass are considered to cause weakness of aortic wall, which can further propagate to coronary arteries (33, 34). High estrogene and progesterone level during pregnancy through decomposition exovascular structural support also may contribute to SCAD (33, 35).

Regarding clinical outcomes, our analysis showed that cutting balloon use in SCAD was associated with a high rate of technical success, as evidenced by satisfactory angiographic results and resolution of coronary flow abnormalities (Supplementary Table 1). The controlled micro-incisions created by cutting balloons seemed to decompress the true lumen and restore adequate blood flow. This may be particularly beneficial in cases of localized dissections or focal stenoses. Moreover, the reported TIMI 3 flow restoration observed in 87.5% of cases supports the potential functional benefits of cutting balloon angioplasty in SCAD patients.

While cutting balloon use demonstrated promising results, it is crucial to acknowledge the potential complications associated with this technique. Our analysis revealed a low incidence of major adverse events, such as coronary perforation, dissection extension, or acute vessel closure. However, it is important to note that in 84.37% of published cases reported a relatively short-term follow-up, limiting our understanding of the long-term outcomes and potential late complications associated with cutting balloon use. Therefore, the safety profile of cutting balloon angioplasty in SCAD warrants further investigation with larger prospective studies and longer-term follow-up.

Furthermore, it is worth highlighting that the existing evidence on cutting balloon use in SCAD is predominantly derived from case reports and small case series, resulting in inherent limitations. The lack of standardized reporting, heterogeneity in procedural techniques, and potential publication bias may limit the generalizability of our findings. Additionally, the absence of a comparative group receiving conventional treatment modalities, such as medical therapy or percutaneous coronary intervention (PCI), hinders our ability to draw definitive conclusions regarding the superiority or inferiority of cutting balloon use in SCAD management.

Despite these limitations, our analysis provides valuable insights into the use of cutting balloons in SCAD and highlights the need for further research in this area. Future studies should focus on larger-scale prospective investigations comparing cutting balloon angioplasty with standard treatment approaches to establish its role in the overall management algorithm for SCAD. Long-term follow-up and comprehensive evaluation of functional outcomes, including exercise capacity and quality of life measures, would help assess the durability of the benefits associated with cutting balloon use.

Conventional PCI for SCAD on the other hand highlights a high periprocedural failure rate and a significant increase in Major Adverse Cardiovascular Events (MACE). The results indicate that PCI was successful in only 34.7% of cases, partially successful in 37.3%, and outright unsuccessful in 28.0%. The propagation of SCAD occurred in 44.0% of cases, and residual dissection was observed in 58.6% of cases. This substantial rate of PCI failures suggests that the conventional approach may not be suitable for a considerable proportion of SCAD patients (36).

Antiplatelet therapy is a fundamental component of drug therapy in SCAD, with 92.3% of patients who underwent CB angioplasty receiving some form of such treatment. Because of the nature of CB employment and subsequent controlled coronary vessel wall damage, antiplatelet therapy is used to prevent platelet aggregation and thrombus formation within the dissected coronary artery. While the use of antiplatelet therapy is widespread in SCAD management, several important considerations deserve attention.

SCAD is a heterogeneous condition, and the choice of antiplatelet therapy should be tailored to each patient's specific presentation. Some SCAD patients may have underlying connective tissue disorders, making them more prone to bleeding complications, while others may require more aggressive platelet inhibition. The use of dual antiplatelet therapy, typically combining aspirin and clopidogrel, was used in 66.66% of patients that received antiplatelet therapy. This approach aims to provide more potent platelet inhibition. However, while it is mandatory for patients who received a stent, it raises concerns about bleeding risk and healing difficulty, particularly in patients who underwent CB angioplasty without stenting. The use of ASA alone was reported in only two cases (17, 30) while and additional two had a relatively short DAPT time of 3 months followed by ASA therapy alone (21). Determining the optimal duration of antiplatelet therapy in SCAD patients remains a challenge. While some patients may benefit from long-term therapy to prevent recurrence, others may face an increased risk of bleeding complications with extended treatment. Clinicians must balance the need for ongoing protection against the risk of adverse events. Regular monitoring of patients on antiplatelet therapy is essential. Platelet function tests, bleeding risk assessments, and coronary imaging may help guide treatment decisions. Close follow-up allows clinicians to adjust therapy based on the patient's response and evolving clinical circumstances.

In addition to the information provided earlier, it's crucial to acknowledge the limited data available regarding the specific type of drug therapy used in SCAD patients. Out of the published cases, only 40.62% reported details about the specific drugs employed in their treatment. This lack of comprehensive data highlights a need for more standardized reporting in SCAD research and a greater emphasis on documenting the types of drug therapies administered.

## 5. Conclusion

In the case of distal, non-occlusive lesions without ongoing ischemia, the consensus is that they should be treated conservatively with prolonged outpatient follow up.

There is still no consensus on optimal treatment when it comes to occlusive proximal lesions with ongoing ischemia, because conventional stenting usually does not provide adequate results (hematoma and dissection propagation.

In conclusion, our analysis of published cases of cutting balloon use in SCAD demonstrates the potential of this intervention in the management of this complex condition. Cutting balloon angioplasty appears to be a technically feasible and safe adjunctive therapy, offering favorable angiographic outcomes and symptomatic relief. However, the limitations inherent in the available evidence necessitate further research to establish the role of cutting balloons in SCAD and optimize patient outcomes.

## Data Availability

The datasets analysed for this study can be found in PubMed, Embase, and Cochrane Library [https://pubmed.ncbi.nlm.nih.gov, https://www.embase.com/landing?status = grey, https://www.cochranelibrary.com].
