# Supplementary material for: An analysis of published cases of cutting balloon use in spontaneous coronary artery dissection
Source: Front Cardiovasc Med. 2023 Nov 9;10:1270530. doi: 10.3389/fcvm.2023.1270530 (PMC10666782; doi:10.3389/fcvm.2023.1270530)
Supplement: Supplementary file 1 [file Table1.docx]

Supplementary Material

# Supplementary Data

Supplementary Material should be uploaded separately on submission. Please include any supplementary data, figures and/or tables.

# Supplementary Figures and Tables

|  | Table 1. An analysis of gathered cases | | | | | | | | | | |
| --- | --- | --- | --- | --- | --- | --- | --- | --- | --- | --- | --- |
| Author | | Sex | Presentation | History of CVD | Artery | Location | Inflation location | Treatment | CB size (mm) | | TIMI before - after |
| Main *et al.* | | F | STEMI | no | DG1 | Medial | Mid DG1 | Cutting balloon angioplasty | | 2,5 x 10 | 0 - 3 |
| Yumoto *et al.* | | F | STEMI | no | LAD | Medial - distal | Mid and dist LAD | Cutting balloon angioplasty | | 2.5 | 0 - 3 |
| McGrath *et al.* | | F | NSTEMI | no | LM - LCx - OM1 | Proximal | Proximal OM1 and proximal LCx | Cutting balloon angioplasty and stenting | | 3,0 x 10 | 3 - 3 |
| Zghouzi *et al.* | | F | STEMI | yes | LAD | Medial | Mid LAD | Cutting balloon angioplasty | | 3,0 x 20 | 0 - 3 |
| Sharma *et al.* | | F | UAP | yes | RCA | Medial | Mid RCA | Cutting balloon angioplasty | | 4,0 x 10 | 2-3 |
| Kaya *et al.* | | F | STEMI | no | LAD | Medial - distal | Mid and dist LAD | Cutting balloon angioplasty | | 2,5 x 10 | 2-3 |
| Matsuura *et al.* | | F | STEMI | yes | LAD | Medial - distal | Dist LAD | Cutting balloon angioplasty | | 3,5; 2,5 | 1-2 |
| Alkhouli *et al.* | | F | NSTEMI-STEMI | yes | LAD | Medial - apical | Mid and dist LAD | Cutting balloon angioplasty and stenting | | 2,0 x 10 | 1-3 |
| Ito *et al.* | | F | STEMI | no | LAD | Medial - distal | Dist LAD | Cutting balloon angioplasty | | 2 | 0-3 |
| Bresson *et al.* | | F | NSTEMI-STEMI | / | LAD | Proximal - medial | Mid LAD | Cutting balloon angioplasty and stenting | | 2,5 x 10 | 1-3 |
| Lee *et al.* | | F | STEMI | no | LAD | Medial | Mid LAD | Cutting balloon angioplasty | | 2,25 x 10 | 1-3 |
| Lee *et al.* | | F | STEMI | no | LAD | Medial - distal | / | Cutting balloon angioplasty and stenting | | 2.5 | [0-1]-3 |
| Uema *et al.* | | F | UAP-STEMI | yes | LAD | Medial - distal | / | Cutting balloon angioplasty | | 3 | 0-3 |
| Noguchi *et al.* | | M | APNS-STEMI | no | LM - LAD - LCx | LM = distal; LAD = Proximal - medial; LCx = Proximal - medial | Mid LCx; Proximal and mid LAD | Cutting balloon angioplasty and stenting | | 3 (LCx); 3,5 (LAD) | LM = 3-3; LAD = 0-3; LCx = 1-3 |
| Bastante *et al.* | | F | UAP | / | LAD | Medial | / | Cutting balloon angioplasty | | 2.5 x 15 | 0-3 |
| Bastante *et al.* | | F | NSTEMI-STEMI | / | LAD | Medial | / | Cutting balloon angioplasty | | 2,5 x 8 | 1-3 |
| Bastante *et al*. | | F | STEMI | / | LAD | Medial | / | Cutting balloon angioplasty | | 2 x 20 | 0-3 |
| Macaya*et al.* | | F | STEMI | / | LAD | Mid-distal | LMS, ostial LAD and LCx | Cutting balloon angioplasty and stenting | | 2,0; 2,5; | 0-3 |
| Fujita *et al.* | | F | NSTEMI | no | LAD | Mid-distal | Mid-distal LAD | Cutting balloon angioplasty | | 2,0x10 | 2-3 |
| Fujita et al. | | F | STEMI | no | RCA | Distal | Dist | Cutting balloon angioplasty | | 2,0x10 | 0-3 |
| Kahata *et al.* | | F | STEMI | no | OM | Distal | Distal OM | Cutting balloon angioplasty | | / | 0-3 |
| Cerrato *et al.* | | F | NSTEMI | no | LAD | Distal | Distal LAD | Cutting balloon angioplasty | | 3,0x10 | 0-3 |
| Mailey *et al.* | | F | STEMI | no | LAD; LCx | Proximal | Proximal - Mid - Distal | Cutting balloon angioplasty and CABG | | 2,5x19; 3,0x10; | 0-2 |
| Yamamoto *et al.* | | M | STEMI | / | LAD | Proximal | Proximal LAD | Cutting balloon angioplasty | | 4,0x15 | 0-3 |
| Choudhury *et al.* | | F | NSTEMI | yes | LAD | Mid - distal | Mid - distal LAD | Cutting balloon angioplasty | | 2,5x10 | [3-0]-3 |
| Somerville *et al.* | | F | NSTEMI | no | LCx - OM | Proximal - Mid (LCx); Proximal (OM) | / | Cutting balloon angioplasty and stenting | | / | 1-3 |
| Ejima *et al.* | | F | STEMI | no | LAD | Mid | / | Cutting balloon angioplasty and stenting | | 3,0x10 | 1-2 (0) |
| Gilpin *et al.* | | F | NSTEMI | no | LAD | Mid | Mid LAD | Cutting balloon angioplasty and stenting | | 3,0 | 1-3 |
| Low *et al.* | | F | STEMI | / | LAD | Proximal | Prox LAD | Cutting balloon angioplasty | | 3.5 | 0-3 |
| McConkey *et al.* | | F | UAP-STEMI | no | LCx | Ostial-proximal | ostial-proximal | Cutting balloon angioplasty and stenting | | 2.5 | 1-3 |
| McConkey et al. | | F | STEMI | no | RCA | Proximal-medial | / | Cutting balloon angioplasty and stenting | | 2.5 | 2-3 |
| Alyamani et al. | | F | SAP | yes | LAD, LCx, RI, RCA | Proximal-distal | prox-mid | Cutting balloon angioplasty and stenting + conservative | | 2,5x10 | 2-3  (2) |
|  | F – female; M – male; CVD – cardiovascular disease; CB – cutting balloon; STEMI – st elevation myocardial infarction; NSTEMI – non st elevation myocardial infarction; UAP – unstable angina pectoris; Dg – diagonal branch; LAD – left anterior descending artery; LCx – lateral cicumflex; RCA – right coronary artery; TIMI - thrombolysis in myocardial infarction | | | | | | | | | | |

## Supplementary Figures


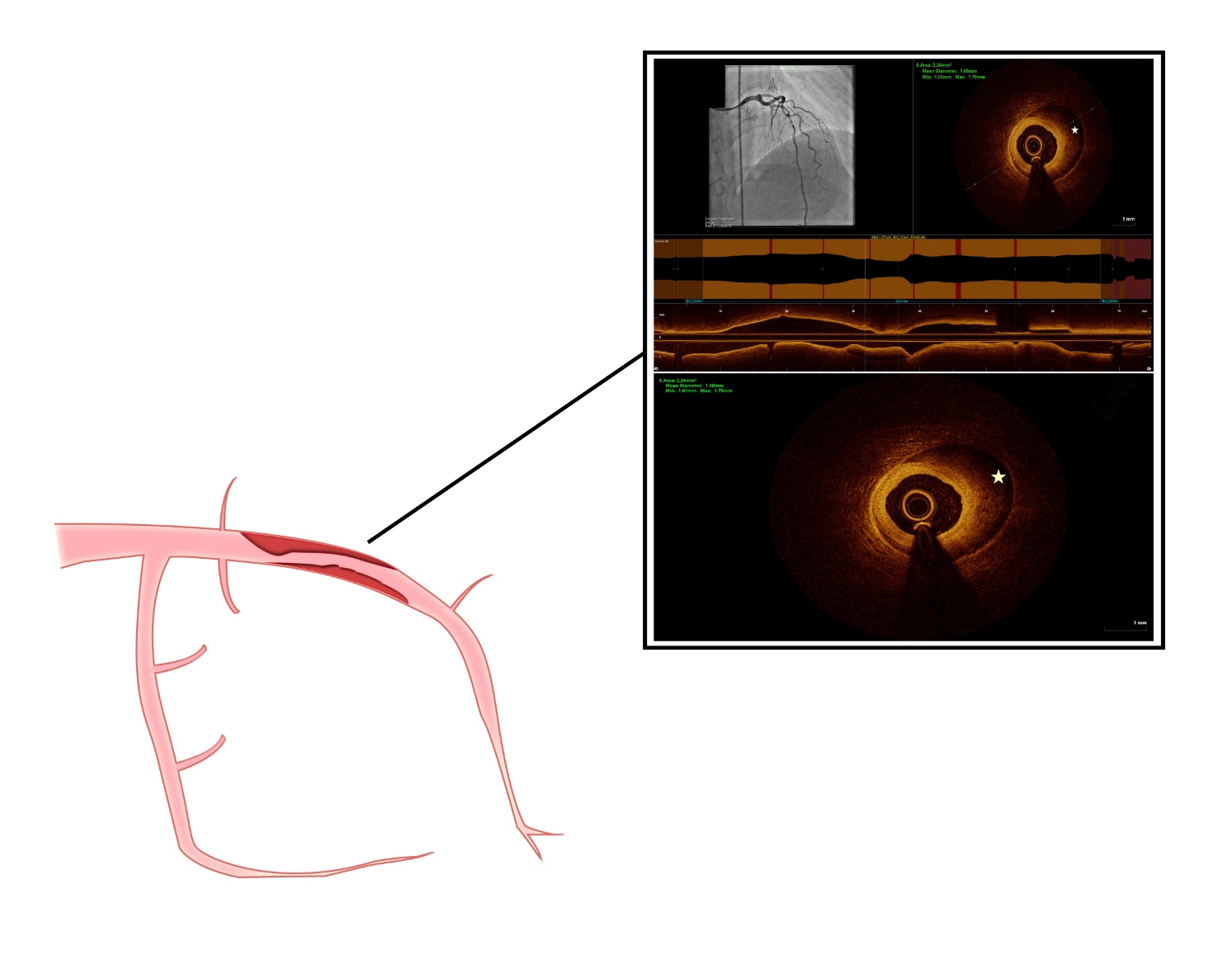


**Figure 1.** A sketch and OCT frames of an intramural hematoma(*) in the mid LAD.


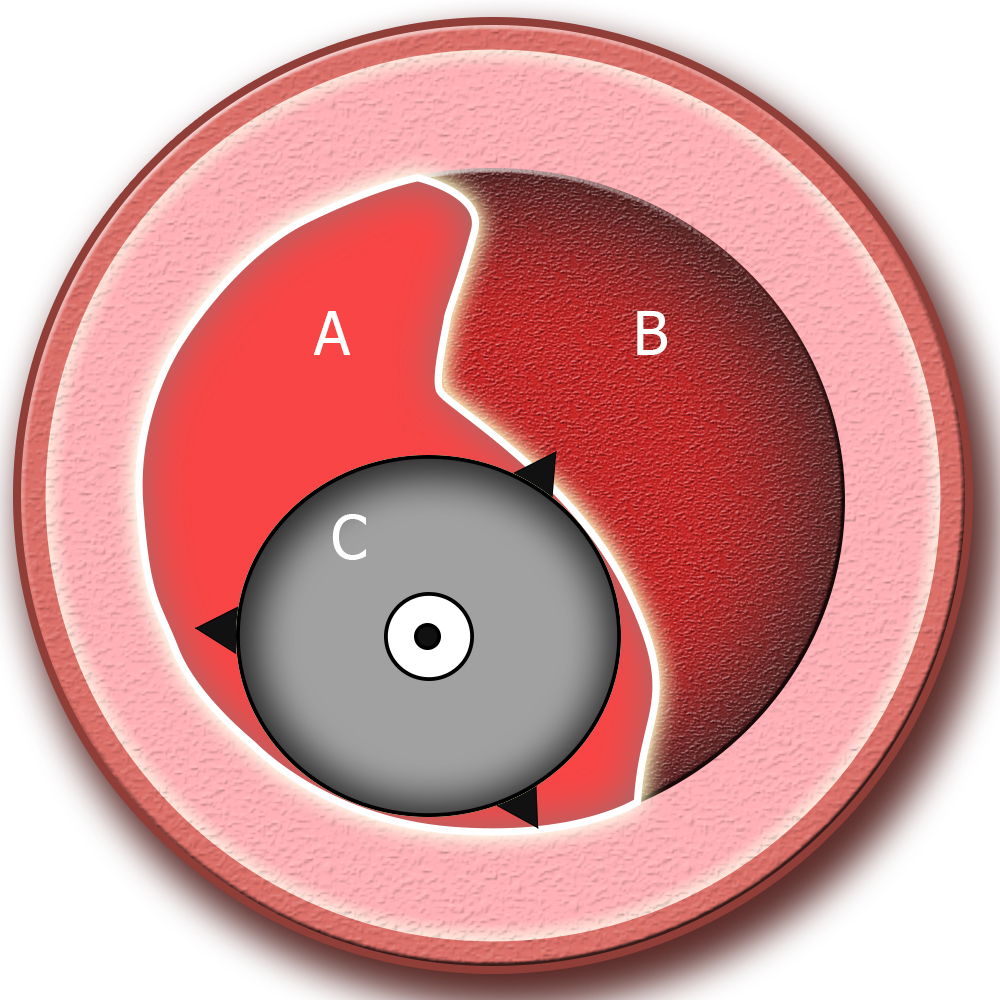


**Figure 2.** A sketch of cutting balloon application inside a vessel with an intramural hematoma: A. True lumen of the blood vessel; B. Faslse lumen (intramural hematoma) causing compression; C. Cutting balloon positioned inside the true lumen in order to cause intimal fenestration.

**
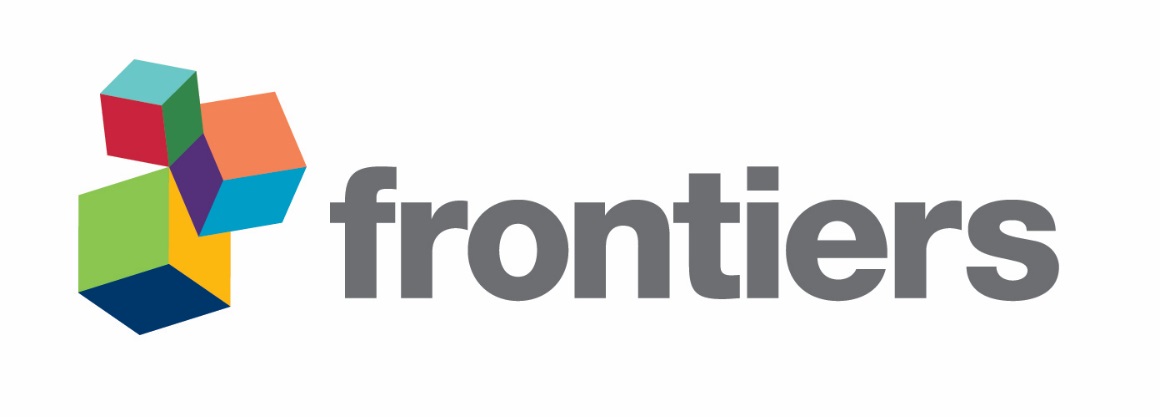
**
